# Focal Amyloidosis of the Orbit Presenting as a Mass: MRI and CT Features

**DOI:** 10.5812/iranjradiol.4555

**Published:** 2011-12-25

**Authors:** Hasan Yerli, Erdinc Aydin, Suat Avci, Nihan Haberal, Sibel Oto

**Affiliations:** 1 Department of Radiology, Zubeyde Hanim Practice and Research Center, Baskent University, Izmir, Turkey; 2 Department of Otolaryngology, Faculty of Medicine, Baskent University, Ankara, Turkey; 3 Department of Pathology, Faculty of Medicine, Baskent University, Ankara, Turkey; 4 Department of Ophthalmology, Faculty of Medicine, Baskent University, Ankara, Turkey

**Keywords:** Amyloidosis, Orbit, Magnetic Resonance Imaging, Tomography, Spiral Computed

## Abstract

Focal orbital amyloidosis is a rare entity and little is known about its magnetic resonance imaging (MRI) features. In this case report, imaging features of a case of focal orbital amyloidosis presenting as a mass have been documented together with its histopathological findings. On MRI, a well-defined mass was seen as isointense with rectus muscle on T1-weighted images and heterogeneously hypointense on T2-weighted images. Punctuate calcifications were observed on the computerized tomography (CT) examination.

## 1. Introduction

Primary orbital amyloidosis is a rare disorder that is seen in the craniocervical region and is formed by focal accumulation of amyloid protein in the orbit. Amyloidosis is classified into two main clinical types; namely, localized and systemic. Although the prognosis of the disease is usually poor in the systemic form, better prognosis is observed in the localized form [1][2][3][4][5][6]. Focal orbital amyloidosis is a very rare disease and less than 200 cases of focal amyloidosis involving the head and neck have been reported in the literature [1][2][3][4][5][6][7][8][9][10][11][12][13][14]. The larynx is affected most frequently and only 4% of focal amyloidosis involving the head and neck have an orbital involvement [1]. Amyloid accumulation is mostly seen in the eyelid, conjunctiva and lacrimal gland [8][10][15]. Extraocular muscle involvement and adjacent bone changes including erosion and hyperostosis are rare [2][8][10][16]. Amyloid depositions may lead to such symptoms as periocular mass (95.8%), ptosis (54.2%), periocular pain or discomfort (25%), proptosis or displacement of the globe (21%), restriction of movement (16.7%) and recurrent subconjunctival hemorrhage (12.5%) [3]. The cause of amyloid accumulation in ocular and orbital tissues is unknown. The plasma cell proliferation and vascular and perivascular amyloidal deposits as the reaction to some immunological processes or foreign objects may play a role in the origination and development of the disease [7].

In the literature, the localized orbital amyloidosis, in which there is no systemic illness, appears in isolated case presentations. The magnetic resonance imaging (MRI) findings as regards focal orbital amyloidosis are little known in the literature [2][17]. On computerized tomography (CT) or MRI, amyloid deposits simulate idiopathic orbital inflammatory disease (pseudotumor), which is the most common cause of an intra-orbital mass lesion in adults and other mass lesions [18]. We present the localized orbital amyloidosis case diagnosed by biopsy following evaluation with CT and MRI.

## 2. Case Presentation

**Figure 1:**
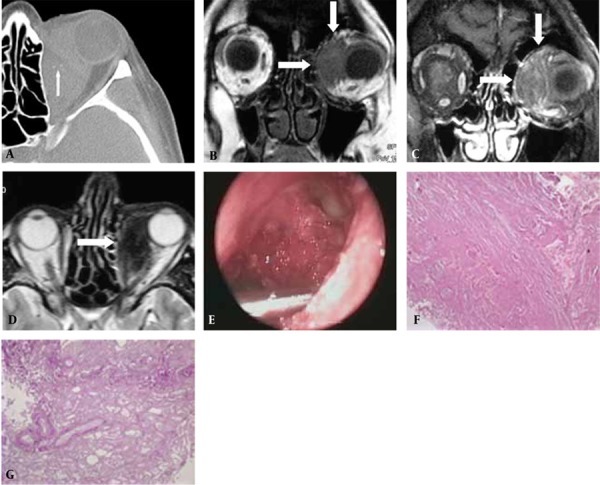
A 60-year-old woman with swelling of the left eye. A, Axial CT scan shows a few small punctate calcific foci (arrow) in a relatively homogeneous soft tissue mass with a well-demarcated margin at the intraconal region of the left orbit. There is minimal expansion in the medial wall of the orbit, and no bone erosion is seen; B, Coronal T1-weighted MR image shows a soft tissue mass (arrows) that is isointense to the rectus muscle. The mass causes lateral displacement of the bulbus oculi and lateral rectus muscle. The medial rectus muscle is not seen at the level of the mass; C, Postcontrast coronal T1-weighted MR image shows heterogeneous enhancement in the soft tissue mass (arrows); D, Axial T2-weighted MR image shows a soft tissue mass (arrow) that is heterogeneously hypointense to the rectus muscle. The mass has caused anterior and lateral displacement of the bulbus oculi; E, An intraoperative photograph shows the soft tissue lesion at the intraconal region of the left orbit; F, Histological section of the lesion shows the homogeneous and amorphous amyloid matrix (Hematoxylin and Eosin × 10); G, Histological section of the lesion shows positive interaction between crystal violet (magnification × 20) and the amyloid accumulation.

A 60-year-old woman was admitted to our clinic with swelling in the left eye. On physical examination; exophthalmus, subconjunctival hemorrhage and restricted eye movements due to a mass localized in the medial side of the orbit were observed. The bulbus oculi was displaced to the anterior and lateral parts of the orbit. On CT examination, a homogeneous soft-tissue mass with regular contours causing minimal expansion in the medial orbit wall was observed at the intraconal region of the left orbit. The mass containing a few small punctate calcific foci (Figure 1A) had dimensions of 4.5 × 4.5 centimeters. On MRI examination, the mass was isointense with the muscle on T1-weighted images (Figure 1B) and showed moderate and heterogeneous contrast enhancement after intravenous contrast material injection (Figure 1C). It was heterogeneously hypointense with the muscle on T2-weighted images. The mass was found to cause medial displacement of the medial rectus muscle and anterior and lateral displacement of the bulbus oculi (Figure 1D).

After biopsy by an endoscopic transnasal and transorbital approach (Figure 1E), the microscopic evaluation was performed. Hematoxylin and eosin stain demonstrated the homogeneous and amorphous amyloid matrix (Figure 1F). The lesion showed positive interaction between crystal violet and amyloid accumulation (Figure 1G). Abdominal ultrasound, electrocardiogram, chest radiography, thyroid function and serum immune electrophoresis tests were normal. Routine blood chemistries, complete blood count, erythrocyte sedimentation rate, antinuclear antibodies, serum angiotensin-converting enzyme, rheumatoid factor and urine laboratory tests were also normal. Rectal biopsy was negative. No finding of systemic amyloidosis was determined during the laboratory investigation and the diagnosis was notified as localized amyloidosis. After 8 weeks following a course of oral prednisone, the lesion was stable. The patient reported mild clinical improvement.

## 3. Discussion

Focal orbital amyloidosis is a relatively uncommon entity and little is known about imaging features at MRI [2][17]. Most of the orbital amyloidosis cases are middle-aged white females. As there is no underlying systemic illness in local amyloidosis, the tests of laboratory and rectal biopsy are found negative. In focal orbital amyloidosis, the amyloid deposits appear usually around the blood vessels in the form of infiltration and they extend towards Tenon’s capsule and orbital and extraocular muscles [3][4][7][8]. Hence, total surgical excision is difficult and its local recurrence is frequent, although the primary treatment is surgical excision. Therefore, management modalities include observation, excision, chemotherapy and steroids depending on the extent of the disease. Radiotherapy may be used to prevent recurrence [19].

The MRI appearances of focal orbital amyloidosis have been reported in a few cases. Okamato et al. reported a case of focal orbital amyloidosis presenting as rectus muscle enlargement [2]. They observed heterogeneous hypointense signals to the muscle on T2-weighted images and homogeneous isointense signals to the muscle on T1-weighted images. Weber et al. reported that the amyloid deposits have similar signal intensities to the muscle on all imaging sequences [17]. Gean-Marton et al. have determined hypointensity in one nasopharyngeal amyloidosis case on T2-weighted images and isointense signals on T1-weighted images [1]. In our case, the orbital amyloidosis appeared as a homogeneous soft tissue mass that was isointense to the muscle on T1-weighted images and heterogeneously hypointense on T2-weighted images (Figure 1D). The low T2-weighted signals for focal amyloidosis have also been observed in different body regions [20][21]. Therefore, it appears that the hypointensity on T2-weighted images is an important clue that supports the diagnosis of focal amyloidosis. The precise mechanism of low T2-weighted signals of amyloidosis is unknown. The amyloid microenvironment including calcification, hemorrhage and microvascular structures may contribute to low T2 signals in focal amyloid deposition.

The presence of punctuate calcifications on CT examination has been determined in eight of 18 cases of orbital amyloidosis [1][2][8][10][22][23]. Mafee et al. demonstrated irregular castlike calcifications involving the retrobulber space, caused by amyloidosis [18]. Adjacent bone erosion, focal thinning or hyperostosis may rarely occur in some cases [8][10]. We observed a few small punctate calcific foci in a relatively homogeneous soft tissue mass with a well-demarcated margin that was isodense with the muscle. In our case, although there was minimal expansion in the orbit medial wall, no bone erosion or hyperostosis was seen. The calcification seen on CT examination (Figure 1A) was not demonstrated on MRI in our case, because, CT is more sensitive than MRI in determining the calcific changes in the lesion content. The CT density of the orbital mass parallel that of the surrounding musculature has been demonstrated [2][18]. The CT findings of our case were in good agreement with previous reports.

The main differential diagnosis of focal orbital amyloidosis based on MRI findings includes pseudotumor, lymphoproliferative lesion, sarcoidosis and cavernous hemangioma. Pseudotumor shows isointense or slightly hyperintense signals to the rectus muscle on T2-weighted images [24]. Nevertheless, they are hypointense compared to many orbital lesions due to cellular infiltration and fibrosis. Pseudotumor shows marked, diffuse and irregular enhancement [25]. A lymphoproliferative lesion is usually mildly hyperintense to the muscle on T2-weighted images. Despite that, lower T2 signals than many other malignancies may be seen due to the cellular nature of lymphoid infiltrate in a lymphoproliferative lesion [25][26]. These lesions show moderate to marked homogeneous enhancement. We think that sarcoidosis cannot be excluded on the basis of MRI only. The low T2 signals can also be seen in a sarcoidosis lesion [27]. However, isolated orbital sarcoidosis is uncommon and is usually limited to the lacrimal gland. Biopsy is often required for the diagnosis of orbital sarcoidosis [27]. Although not pathognomonic, the MRI findings, which suggest cavernous hemangioma, are high signal intensity on T2-weighted images and pseudocapsule that may be seen as a hypointense rim on T1-weighted images [28].

In conclusion, focal orbital amyloidosis that appears as a rare condition among middle-aged females should be kept in mind in the differential diagnosis of orbital masses. The presence of heterogeneous hypointensity on T2-weighted images on MRI and punctuate calcifications on CT examination may be the important findings supporting the diagnosis of focal orbital amyloidosis.
